# What’s Decidable About Program Verification Modulo Axioms?

**DOI:** 10.1007/978-3-030-45237-7_10

**Published:** 2020-03-13

**Authors:** Umang Mathur, P. Madhusudan, Mahesh Viswanathan

**Affiliations:** 8 Johannes Kepler University, Linz, Austria; 9 University of Birmingham, Birmingham, UK; University of Illinois, Urbana Champaign, USA

## Abstract

We consider the decidability of the verification problem of programs *modulo axioms* — automatically verifying whether programs satisfy their assertions, when the function and relation symbols are interpreted as arbitrary functions and relations that satisfy a set of first-order axioms. Though verification of uninterpreted programs (with no axioms) is already undecidable, a recent work introduced a subclass of *coherent* uninterpreted programs, and showed that they admit decidable verification [26]. We undertake a systematic study of various natural axioms for relations and functions, and study the decidability of the coherent verification problem. Axioms include relations being reflexive, symmetric, transitive, or total order relations, functions restricted to being associative, idempotent or commutative, and combinations of such axioms as well. Our comprehensive results unearth a rich landscape that shows that though several axiom classes admit decidability for coherent programs, coherence is not a panacea as several others continue to be undecidable.

### 1 Introduction

Programs are proved correct against safety specifications typically by induction—the induction hypothesis is specified using *inductive invariants* of the program, and one proves that the reachable states of the program stays within the region defined by the invariants, inductively. Though there has been tremendous progress in the field of *decidable logics* for proving that invariants are inductive, finding inductive invariants is almost never fully automatic. And completely automated verification of programs is almost always undecidable.

Programs can be viewed as working over a data-domain, with variables storing values over this domain and being updated using constants, functions and relations defined over that domain. Apart from the notable exception of finite data domains, program verification is typically undecidable when the data domain is infinite. In a recent paper, Mathur et. al. [26] establish new decidability results when the data domain is infinite. Two crucial restrictions are imposed — data domain functions and relations are assumed to be *uninterpreted* and programs are assumed to be *coherent* (the meaning of coherence is discussed later

* Umang Mathur is partially supported by a Google PhD Fellowship. P. Madhusudan is partially supported by NSF CCF 1527395. Mahesh Viswanathan is partially supported by NSF CCF 1901069

in this introduction). The theory of uninterpreted functions is an important theory in SMT solvers that is often used (in conjunction with other theories) to solve feasibility of loop-free program snippets, in bounded model-checking, and to validate verification conditions. The salient aspect of [26] is to show that entire program verification is decidable for the class of coherent programs, without any user-provided inductive invariants (like loop invariants). While the results of [26] were mainly theoretical, there has been recent work on applying this theory to verifying memory-safety of heap-manipulating programs [28].

Data domain functions and relations used in a program usually satisfy special properties and are not, of course, entirely uninterpreted. The results of [26] can be seen as an approximate/abstraction-based verification method in practice — if the program verifies assuming functions and relations to be uninterpreted, then the program is correct for *any* data domain. However, properties of the data domain are often critical in establishing correctness. For example, in order to prove that a sorting program results in sorted arrays, it is important that the binary relation $<$ used to compare elements of the array is a total ordering on the underlying data sort. Consequently, constraining the data domain to satisfy certain axioms results in more accurate modeling for verification.

*In this paper, we undertake a systematic study of the verification of uninterpreted programs when the data-domains are constrained using theories specified by (universally quantified) axioms.* The choice of the axioms we study are guided by two principles. First, we study natural mathematical properties of functions and relations. Second, we choose to study axioms that have a decidable *quantifier-free* fragment of first order logic. The reason is that even single program executions (as defined in Section 3.2) can easily encode quantifier-free formulae (by computing the terms in variables, and assert Boolean combinations of atomic relations and equality on them). Since we are seeking decidable verification for programs *with loops/iteration*, it makes little sense to examine axioms where even verification of single executions is undecidable.

**Coherence modulo theories:** Mathur et. al. [26] define a subclass of programs, called *coherent programs*, for which program verification on uninterpreted domains is decidable; without the restriction of *coherence*, program verification on uninterpreted domains is undecidable. Since our framework is strictly more powerful, we adapt the notion of coherence to incorporate theories. A coherent program [26] is one where all executions satisfy two properties — memoizing and early-assumes. The memoizing property demands that the program computes any term, modulo congruence induced by the equality assumes in the execution, only once. More precisely, if an execution recomputes a term, the term should be stored in a current variable. The early-assumes restriction demands, intuitively, that whenever the program assumes two terms to be equal, it should do so early, before computing superterms of them.

We adapt the above notion to *coherence modulo theories*[1]. The memoizing and early-assumes property are now required modulo the equalities that are entailed by the axioms. More precisely, if the theory is characterized by a set of axioms $\mathcal{A}$, the memoizing property demands that if a program computes a term $t$ and there was another term $t'$ that it had computed earlier which is equivalent to $t$ modulo the assumptions made thus far *and the axioms* $\mathcal{A}$, then $t'$ must be currently stored in a variable. Similarly, the early-assumes condition is also with respect to the axioms — if the program execution observes a new assumption of equality or a relation holding between terms, then we require that any equality entailed newly by it, the previous assumptions *and the axioms* $\mathcal{A}$ do not involve a dropped term. This is a smooth extension of the notion of coherence from [26]; when $\mathcal{A} = \varnothing$, we essentially retrieve the notion from [26].

### Main Contributions

Our first contribution is an extension of the notion of coherence in [26] to handle the presence of axioms, as described above; this is technically nontrivial and we provide a natural extension.

Under the new notion of coherence, we first study axioms on relations. The EPR (effectively propositional reasoning) [37] fragment of first order logic is one of the few fragments of first order logic that is decidable, and has been exploited for bounded model-checking and verification condition validation in the literature [34,33,32]. We study axioms written in EPR (i.e., universally quantified formulas involving only relations) and show that verification for even coherent programs, modulo EPR axioms, is undecidable.

Given the negative result on EPR, we look at particular natural axioms for relations, which are nevertheless expressible in EPR. In particular, we look at reflexivity, irreflexivity, and symmetry axioms, and show that verification of coherent programs is decidable when the interpretation of some relational symbols is constrained to satisfy these axioms. Our proof proceeds by instrumenting the program with auxiliary `assume` statements that preserve coherence and subtle arguments that show that verification can be reduced to the case without axioms; decidability then follows from results established in [26].

We then show a much more nontrivial result that verification of coherent programs remains decidable when some relational symbols are constrained to be transitive. The proof relies on new automata constructions that compute streaming congruence closures while interpreting the relations to be transitive.

Furthermore, we show that combinations of reflexivity, irreflexivity, symmetry, and transitivity, admit a decidable verification problem for coherent program. Using this observation, we conclude decidability of verification when certain relations are required to be strict partial orders (irreflexive and transitive) or equivalence relations.

[1] We adapt the definition in a way that preserves the spirit of the definition of coherence. Moreover, if we do not adapt the definition, essentially all axioms classes we study in this paper would be undecidable.

We then consider axioms that capture total orders and show that they too admit a decidable coherent verification problem. Total orders are also expressible in EPR and their formulation in EPR has been used in program verification, as they can be used in lieu of the ordering on integers when only ordering is important. For example, they can be used to model data in sorting algorithms, array indices in modeling distributed systems to model process ids and the states of processes, etc. [34,33].

Our next set of results consider axioms on functions. Associativity and commutativity are natural and fundamental properties of functions (like + and ∗) and are hence natural ways to capture/abstract using these axioms. (See [14] where such abstractions are used in program analysis.) We first show that verification of coherent programs is decidable when some functions are assumed to be commutative or idempotent. Our proof, similar to the case of reflexive and symmetric relations, relies on reducing verification to the case without axioms using program instrumentation that capture the commutativity and idempotence axioms. However, when a function is required to be associative, the verification problem for coherent programs becomes undecidable. This undecidability result was surprising to us.

The decidability results established for properties of individual relation or function symbols discussed above can be combined to yield decidable verification modulo a set of axioms. That is, the verification of coherent programs with respect to models where relational symbols satisfy some subset of reflexivity/irreflexivity/symmetery/transitivity axioms or none, and function symbols are either uninterpreted, commutative, or idempotent, is decidable.

Decidability results outlined above, apply to programs that are coherent modulo the axioms/theories. However, given a program, in order to verify it using our techniques, we would also like to decide whether the program *is* coherent modulo axioms. We prove that for all the decidable axioms above, checking whether programs are coherent modulo the axioms is a decidable problem. Consequently, under these axioms, we can both check whether programs are coherent modulo the axioms and if they are, verify them.

There are several other results that we mention only in passing. For instance, we show that even for single executions, verifying them modulo equational axioms is undecidable as it is closely related to the word problem for groups. And our positive results for program verification under axioms for functions (commutativity, idempotence), also shows that bounded model-checking under such axioms is decidable, which can have its own applications.

Due to the large number of results and technically involved proofs, we give only the main theorems and proof gists for some of these in the paper; details can be found in [27].

### 2 Illustrative Example

Consider the problem of searching for an element `k` in a sorted list. There are two simple algorithms for this problem. Algorithm 1 walks through the list from

```
assume (T ≠ F);
found := F;
stop := F;
exists := F;
sorted := T;
while( x ≠ NIL) {
  if( stop = F) then {
    if( k = key(x)) then found := T;
    if( k ≤ key(x)) then stop := T;
  }
  if( k = key(x)) then exists := T;
  y := next(x);
  if( y ≠ NIL) then {
    if( k(x) ≰ k(y)) then sorted := F;
  }
  x := y;
}
@post: sorted = T ⟹ found = exists
```

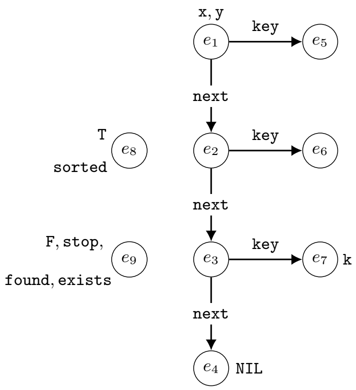

$<$ is $\{(e_5, e_6), (e_6, e_7), (e_7, e_5)\}$

**Fig. 1.** Left: Uninterpreted program for finding a key k in a list starting at x with $<$ interpreted as a strict total order. The condition $a \leq b$ is shorthand for $a < b \vee a = b$. Right: A model in which $<$ is not interpreted as a strict total order. The elements in the universe of the model are denoted using circles. Some elements are labeled with variables denoting the initial values of these variables. The edges ⟶ represent subterm relation. Not all functions are shown in the figure. The model does not satisfy the post-condition on the program on left.

beginning to end, and if it finds `k`, it sets a Boolean variable `exists` to `T`. Notice this algorithm does not exploit the sortedness property of the list. Algorithm 2 also walks through the list, but it stops as soon as it either finds `k` or reaches an element that is larger than `k`. If it finds the element it sets a Boolean variable `found` to `T`. If both algorithms are run on the same sorted list, then their answers (namely, `exists` and `found`) must be the same.

Fig. 1 (on the left) shows a program that weaves the above two algorithms together (treating Algorithm 1 as the specification for Algorithm 2). The variable `x` walks down the list using the `next` pointer. The variable `stop` is set to `T` when Algorithm 2 stops searching in the list. The precondition, namely that the input list is sorted, is captured by tracking another variable `sorted` whose value is `T` if consecutive elements are ordered as the list is traversed. The post condition demands that whenever the list is sorted, `found` and `exists` be equal when the list has been fully traversed. Note that the program's correctness is specified using only quantifier-free assertions using the same vocabulary as the program.

The program works on a data domain that provides interpretations for the functions `key`, `next`, the initial values of the variables, and the relation $<$. When $<$ is interpreted to be a strict total order, the program is correct. However, if $<$ is not interpreted as a total order, then the program may be incorrectly deemed as buggy. To see this, consider the data model shown on the right in Fig. 1. The data domain has 9 elements in its universe, with the functions `next` and `key` interpreted as shown. Initially, `x, y` have value $e_1$, `NIL` is $e_4$, `k` is $e_7$, `T` and `sorted` are $e_8$, and `F, found, exists`, and `stop` are $e_9$. The interpretation of $<$ is as follows — $e_5 < e_6$, $e_6 < e_7$, and $e_7 < e_5$. Clearly $<$ is not an order, but the program's *sortedness* check "`sorted` = `T`" will pass. After the entire list is processed, `exists` will be set to `T` when `x` $= e_3$. On the other hand, `stop` will be set to `T` when `x` $= e_1$ because `k` $= e_7 <$ `key(x)`. Therefore, at the end `found` = `F` $\neq$ `exists`. The work presented in [26], where all functions and relations are uninterpreted, would therefore declare this program to be incorrect.

The goal of this paper is to explore several natural restrictions on data models and study the problem of verifying coherent programs for them. When $<$ is constrained to be a total order, the program in Fig. 1 is correct and coherent. Our results (see Section 5.5) show that verification of such programs when relations are constrained to be strict total orders is decidable, and hence we can build automatic decision procedures that will correctly verify such programs.

### 3 Preliminaries

We briefly recall the syntax and semantics of uninterpreted programs and the verification problem modulo axioms. Our presentation closely follows [26] and for lack of space, some details have been postponed to [27].

#### 3.1 Program Syntax

We consider imperative programs with loops over a fixed finite set of variables $V$ and use constant ($\mathcal{C}$), function ($\mathcal{F}$), and predicate ($\mathcal{R}$) symbols belonging to some first order signature $\Sigma = (\mathcal{C}, \mathcal{F}, \mathcal{R})$. Programs are then given by the syntax below ($f \in \mathcal{F}, R \in \mathcal{R}$, $x, y \in V$, $\mathbf{z}$ is a tuple of variables in $V$):

$$
\begin{aligned}
\langle \text{stmt} \rangle ::= &\mid x := y \mid x := f(\mathbf{z}) \mid \textbf{assume}\,(\langle \text{cond} \rangle) \mid \textbf{skip} \mid \langle \text{stmt} \rangle \,;\, \langle \text{stmt} \rangle \\
&\mid \textbf{while}\,(\langle \text{cond} \rangle)\,\langle \text{stmt} \rangle \mid \textbf{if}\,(\langle \text{cond} \rangle)\,\textbf{then}\,\langle \text{stmt} \rangle\,\textbf{else}\,\langle \text{stmt} \rangle \\
\langle \text{cond} \rangle ::= &\, x = y \mid R(\mathbf{z}) \mid \neg \langle \text{cond} \rangle
\end{aligned}
$$

#### 3.2 Executions and Semantics of Uninterpreted Programs

Executions of programs over $\langle \text{stmt} \rangle$ are words over the following alphabet

$$
\begin{aligned}
\Pi = \{&\text{“}x := y\text{”}, \text{“}x := f(\mathbf{z})\text{”}, \text{“}\textbf{assume}(x = y)\text{”}, \text{“}\textbf{assume}(x \neq y)\text{”}, \\
&\text{“}\textbf{assume}(R(\mathbf{z}))\text{”}, \text{“}\textbf{assume}(\neg R(\mathbf{z}))\text{”} \mid x, y \in V, \mathbf{z} \textit{ is in tuples}(V)\}
\end{aligned}
$$

For a program $s \in \langle \text{stmt} \rangle$, the set of executions of $s$, denoted $\mathsf{Exec}(s)$ is a regular language over the alphabet $\Pi$ and is given as follows (similar to [26]).

$$
\begin{aligned}
&\mathsf{Exec}(\textbf{skip}) = \epsilon && \mathsf{Exec}(x := y) = \text{“}x := y\text{”} \\
&\mathsf{Exec}(x := f(\mathbf{z})) = \text{“}x := f(\mathbf{z})\text{”} && \mathsf{Exec}(\textbf{assume}(c)) = \text{“}\textbf{assume}(c)\text{”}
\end{aligned}
$$

$$
\begin{aligned}
\mathsf{Exec}(\textbf{if } c \textbf{ then } s_1 \textbf{ else } s_2) &= \text{“}\textbf{assume}(c)\text{”} \cdot \mathsf{Exec}(s_1) + \text{“}\textbf{assume}(\neg c)\text{”} \cdot \mathsf{Exec}(s_2) \\
\mathsf{Exec}(s_1; s_2) &= \mathsf{Exec}(s_1) \cdot \mathsf{Exec}(s_2) \\
\mathsf{Exec}(\textbf{while } c\ \{s\}) &= [\text{“}\textbf{assume}(c)\text{”} \cdot \mathsf{Exec}(s_1)]^* \cdot \text{“}\textbf{assume}(\neg c)\text{”}
\end{aligned}
$$

The set of partial executions of $s$ is the set of prefixes of words in $\mathsf{Exec}(s)$ and is also regular.

A data model $\mathcal{M} = (U_{\mathcal{M}}, [\![]\!]_{\mathcal{M}})$ for signature $\Sigma$ is a first order structure where $U_{\mathcal{M}}$ is a universe of elements and $[\![]\!]_{\mathcal{M}}$ maps every symbol in $\Sigma$ to their interpretations. Given a data model $\mathcal{M}$ over $\Sigma$, and an execution $\rho \in \Pi^*$, the semantics of $\rho$ on $\mathcal{M}$ is given by $\mathsf{eval}_{\mathcal{M}} : \Pi^* \times V \to U_{\mathcal{M}}$ that gives the the valuation of variables in $V$ at the end of an execution; the precise definition is standard and is defered to [27].

#### 3.3 Feasibility of Executions Modulo Axioms

An execution is said to be *feasible* in a data model, if every assumption made in the execution, holds on the model. More precisely, an execution $\rho$ is feasible in $\mathcal{M}$ if for every prefix $\sigma' = \sigma \cdot$ "**assume** $c$" of $\rho$, we have

(a) $\mathsf{eval}_{\mathcal{M}}(\sigma, x) = \mathsf{eval}_{\mathcal{M}}(\sigma, y)$ if $c$ is '$(x = y)$',
(b) $\mathsf{eval}_{\mathcal{M}}(\sigma, x) \neq \mathsf{eval}_{\mathcal{M}}(\sigma, y)$ if $c$ is '$(x \neq y)$',
(c) $(\mathsf{eval}_{\mathcal{M}}(\sigma, z_1), \ldots, \mathsf{eval}_{\mathcal{M}}(\sigma, z_r)) \in [\![R]\!]_{\mathcal{M}}$ if $c$ is '$R(z_1, \ldots, z_r)$', and
(d) $(\mathsf{eval}_{\mathcal{M}}(\sigma, z_1), \ldots, \mathsf{eval}_{\mathcal{M}}(\sigma, z_r)) \notin [\![R]\!]_{\mathcal{M}}$ if $c$ is '$\neg R(z_1, \ldots, z_r)$'.

Let $\mathcal{A}$ be a set of first order sentences, including possible ground atomic predicates [2]. We say that a data model $\mathcal{M}$ is an $\mathcal{A}$-model, denoted $\mathcal{M} \models \mathcal{A}$, if for every $\varphi \in \mathcal{A}$, we have $\mathcal{M} \models \varphi$. A formula $\varphi$ is $\mathcal{A}$-valid, denoted $\mathcal{A} \models \varphi$, if $\phi$ holds in every model $\mathcal{M}$ that satisfies $\mathcal{A}$. An execution $\rho$ is said to be *feasible modulo* $\mathcal{A}$ if there is an $\mathcal{A}$-model $\mathcal{M}$ such that $\rho$ is feasible in $\mathcal{M}$.

#### 3.4 Program Verification Modulo Axioms

We consider programs annotated with post-conditions that are over the following syntax below. Here, $x, y$ and $\mathbf{z}$ belong to the set of program variables $V$ and $R \in \mathcal{R}$ is a relation symbol in $\Sigma$.

$$\mathcal{L}: \qquad \varphi ::= x{=}y \mid R(\mathbf{z}) \mid \varphi \vee \varphi \mid \neg\varphi$$

**Definition 1 (Program Verification Modulo Axioms).** *For a program $s$ and a set of axioms $\mathcal{A}$, we say that $s$ satisfies a postcondition $\varphi$ over the syntax $\mathcal{L}$* modulo $\mathcal{A}$ *if for every $\mathcal{A}$-model $\mathcal{M}$ and for execution $\rho \in \mathsf{Exec}(s)$ that is feasible in $\mathcal{M}$, $\mathcal{M}$ satisfies $\varphi[\mathsf{eval}_{\mathcal{M}}(\rho, V)/V]$ (i.e., where each variable $x \in V$ is replaced by $\mathsf{eval}_{\mathcal{M}}(\rho, V)$).*

We remark that one can alternatively phrase the verification problem stated above in terms of feasibility. That is, a program $s$ satisfies a postcondition $\varphi$ modulo $\mathcal{A}$ iff every execution $\rho$ of $s'$ is infeasible modulo $\mathcal{A}$ (i.e., there is no $\mathcal{A}$-model $\mathcal{M}$ such that $\rho$ is feasible in $\mathcal{M}$), where $s' = s;\mathbf{assume}(\neg\varphi)$.

[2] A ground atomic predicate is of the form $t_1 \sim t_2$, or $R(t_1, \ldots t_k)$ or $\neg R(t_1, \ldots t_k)$, where $\sim \in \{=, \neq\}$, $R$ is a relation symbol, and $t_i$s are ground terms.

## 4 Coherence Modulo Axioms

In this section we extend the notion of coherence from [26], adapting it to our current setting where we restrict data models using axioms $\mathcal{A}$. We will first recall the notion of terms computed by an execution, which will be used to define the notion of coherence.

### 4.1 Terms Computed and Assumptions Accumulated by Executions

We will associate a syntactic term $\mathsf{TEval}(\rho, x)$ with each variable $x \in V$ after a partial execution $\rho$. Intuitively, every variable $x \in V$ stores a constant term $\widehat{x}$ in the beginning of an execution. New terms are computed on function computations, i.e., $\mathsf{TEval}(\rho \cdot \text{“}x := f(z_1, \ldots, z_r)\text{”}) = f(\mathsf{TEval}(\rho, z_1), \ldots, \mathsf{TEval}(\rho, z_r))$. The precise definition is simple and is defered to [27]. The set of terms computed by an execution $\rho$ is $\mathsf{Terms}(\rho) = \{\mathsf{TEval}(\rho', x) \mid \rho' \text{ is a prefix of } \rho, x \in V\}$.

As an execution proceeds, it accumulates assumptions over the terms it computes, and we will use $\kappa(\rho)$ to denote the assumptions made by the execution $\rho$ (see [27] for precise definition). For example, after an equality assume statement “**assume**$(x = y)$”, we accumulate the atomic equality predicate $\psi = t_x = t_y$, where $t_x$ and $t_y$ are terms associated with $x$ and $y$ when the assume statement is encountered. Similarly, for the execution $\rho = \rho' \cdot$ “**assume**$(\neg R(z_1, z_2, \ldots, z_k))$”, we have $\kappa(\rho) = \kappa(\rho') \cup \{\neg R(\mathsf{TEval}(\rho', z_1), \ldots, \mathsf{TEval}(\rho', z_k))\}$.

### 4.2 Coherence

Our definition of coherence modulo axioms is a smooth generalization of the definition of coherence in [26]. The notion of coherence consists of two properties — *memoizing* and *early equality assumes*. The memoizing property says, intuitively, when a term $t$ is computed after executing some prefix $\sigma$ of an execution, if $t$ is equivalent to some other term modulo the assumptions made in the execution so far, then $t$ must not have been *dropped* at the end of $\sigma$, i.e., a program variable must already hold this term. We replace the notion of equivalence of terms in this definition by equivalence modulo the axioms as well.

The notion of early assumes in [26] intuitively says that assumptions of equality (on terms $t_1$ and $t_2$) should be encountered early — earlier than *dropping* any superterm of $t_1$ or $t_2$. This notion of early assumes allows for effectively computing *congruence closure* on the set of terms computed by the execution, which in turn, is necessary to accurately maintain which terms are equivalent. However, we observe that the notion in [26] is too restrictive and not entirely necessary. In our paper, we generalize this notion in several ways, to a more semantic one as follows. Whenever an execution encounters an assumption of equality between two term, we instead demand that only the equivalences that are *additionally* implied by this new assumption, can be infered *locally* using the already known congruence between terms in the *window*, i.e., the set of terms pointed to by the program variables when the equality assumption is encountered. Next, we incorporate axioms into this definition, by requiring that the notion of equivalence is also modulo the axioms, and further require that *all* assumptions (equality, disequality, relational) are required to be early (as against only restricting equality assumptions to be early like in [26]). We will elaborate on these differences using an example after presenting the formal definition next.

Given a set of first order sentences $\Gamma$ and ground terms $t_1$ and $t_2$, we say that $t_1 \cong_\Gamma t_2$ if $\Gamma \models t_1 = t_2$.

**Definition 2 (Coherence modulo axioms).** *Let $\mathcal{A}$ be a set of axioms and let $\rho$ be a complete or partial execution over variables $V$. Then, $\rho$ is said to be coherent modulo $\mathcal{A}$ if it satisfies the following two properties.*

**Memoizing.** *Let $\pi = \sigma \cdot$ "$x{:=}f(\mathbf{z})$" be a prefix of $\rho$ and let $t = \mathsf{TEval}(\pi, x)$. If there is a term $t' \in \mathsf{Terms}(\sigma)$ such that $t' \cong_{\mathcal{A}\cup\kappa(\sigma)} t$, then there must exist some variable $y \in V$ such that $\mathsf{TEval}(\sigma, y) \cong_{\mathcal{A}\cup\kappa(\sigma)} t$.*

**Early Assumes.** *Let $\pi = \sigma \cdot$ "**assume**$(c)$" be a prefix of $\rho$, where $c$ is any of $x{=}y$, $x{\neq}y$, $R(\mathbf{z})$, or $\neg R(\mathbf{z})$. Let $t \in \mathsf{Terms}(\sigma)$ be a term computed in $\sigma$ such that $t$ has been* dropped*, i.e., for every $x \in V$, we have $\mathsf{TEval}(\sigma, x) \not\cong_{\mathcal{A}\cup\kappa(\sigma)} t$. For any term $t' \in \mathsf{Terms}(\sigma)$, if $t \cong_{\mathcal{A}\cup\kappa(\pi)} t'$, then $t \cong_{\mathcal{A}\cup\kappa(\sigma)} t'$.*

**Remark.** We remark that every execution that is coherent as per the definition in [26], is also coherent modulo $\mathcal{A} = \varnothing$ as in Definition 2. However, the converse is not true and we illustrate this difference below.

*Example 1.* Let us now illustrate the notion of coherence in the presence of axioms using the execution $\rho$ below.

$$\rho = \mathtt{z_1} := \mathtt{f}(\mathtt{x}, \mathtt{y}) \cdot \mathtt{z_2} := \mathtt{f}(\mathtt{y}, \mathtt{x}) \cdot \mathtt{z_3} := \mathtt{g}(\mathtt{z_1}) \cdot \mathtt{z_4} := \mathtt{g}(\mathtt{z_2}) \cdot \mathtt{z_3} := \mathtt{z_5} \cdot \mathtt{z_6} := \mathtt{g}(\mathtt{z_1})$$

Let $\rho_i$ denote the prefix of $\rho$ of length $i$. Here, $\mathsf{TEval}(\rho_3, \mathtt{z_3}) = \mathtt{g}(\mathtt{f}(\widehat{\mathtt{x}}, \widehat{\mathtt{y}}))$, $\mathsf{TEval}(\rho_5, \mathtt{z_3}) = \widehat{\mathtt{z_5}} \neq \mathtt{g}(\mathtt{f}(\widehat{\mathtt{x}}, \widehat{\mathtt{y}}))$ and $\mathsf{TEval}(\rho_6, \mathtt{z_6}) = \mathtt{g}(\mathtt{f}(\widehat{\mathtt{x}}, \widehat{\mathtt{y}}))$. When the set of axioms is $\mathcal{A} = \varnothing$, this execution is not coherent modulo $\mathcal{A}$ as it violates the memoizing requirement at the last statement $\mathtt{z_6} := \mathtt{g}(\mathtt{z_1})$ (no variable stores the term $\mathtt{g}(\mathtt{f}(\widehat{\mathtt{x}}, \widehat{\mathtt{y}}))$ after $\rho_5$).

Now, consider the axiom set denoting commutativity of $\mathtt{f}$, i.e., $\mathcal{A}_{\mathsf{comm}} = \{\forall u, v.\mathtt{f}(u, v) = \mathtt{f}(u, v)\}$. In this case, we observe that $\mathtt{f}(\widehat{\mathtt{x}}, \widehat{\mathtt{y}}) \cong_{\mathcal{A}_{\mathsf{comm}}} \mathtt{f}(\widehat{\mathtt{y}}, \widehat{\mathtt{x}})$ and thus $\mathtt{g}(\mathtt{f}(\widehat{\mathtt{x}}, \widehat{\mathtt{y}})) \cong_{\mathcal{A}_{\mathsf{comm}}} \mathtt{g}(\mathtt{f}(\widehat{\mathtt{y}}, \widehat{\mathtt{x}}))$. Also, $\mathsf{TEval}(\rho_5, \mathtt{z_4}) = \mathtt{g}(\mathtt{f}(\widehat{\mathtt{y}}, \widehat{\mathtt{x}})) \cong_{\mathcal{A}_{\mathsf{comm}}} \mathtt{g}(\mathtt{f}(\widehat{\mathtt{x}}, \widehat{\mathtt{y}}))$. This ensures that $\rho$ is indeed coherent modulo $\mathcal{A}_{\mathsf{comm}}$.

Let $\mathsf{CoherentExecs}(\Sigma, V, \mathcal{A})$ denote the set of executions over the signature $\Sigma$ and variables $V$ that are coherent modulo the set of axioms $\mathcal{A}$.

**Definition 3.** *A program $s$ over signature $\Sigma$ and variables $V$ is said to be coherent modulo $\mathcal{A}$ if $\mathsf{Exec}(s) \subseteq \mathsf{CoherentExecs}(\Sigma, V, \mathcal{A})$.*

In this paper, we explore several classes of axioms, studying when the verification problem for coherent programs modulo the axioms is decidable.

## 5 Axioms over Relations

In this section, we investigate the decidability of the verification problem for coherent programs modulo relational axioms, i.e., axioms which only involve relation symbols $\mathcal{R}$ in the signature $\Sigma$.

### 5.1 Verification modulo EPR axioms

A first-order formula is said to be an EPR formula [37] if it is of the form

$$\exists x_1 \dots x_k \forall y_1, \dots y_m \; \varphi$$

where $\varphi$ is quantifier-free and purely relational (uses no function symbols).

It is well known that satisfiability of EPR formulas is decidable, in fact by a reduction to Boolean satisfiability [24]. Consequently, the problem of checking whether a single execution is feasible under axioms written in EPR can be shown to be decidable, and has been exploited in bounded model-checking.

Consequently, we could reasonably ask whether verification of coherent programs under EPR axioms is decidable. Surprisingly, we show that they are not (proof details can be found in [27]).

**Theorem 1.** *Verification of uninterpreted coherent programs modulo EPR axioms is undecidable.* □

Given the above result, we turn to several classes of quantified axioms, which are all expressible in EPR (and hence have a decidable bounded model checking problem) and examine their decidability for coherent program verification.

### 5.2 Reflexivity, Irreflexivity, and Symmetry

We consider program verification under the following axioms (individually):

$$\begin{array}{lll} \varphi^R_{\mathsf{refl}} & \triangleq \forall x \cdot R(x,x) & \text{(reflexivity)} \\ \varphi^R_{\mathsf{irref}} & \triangleq \forall x \cdot \neg R(x,x) & \text{(irreflexivity)} \\ \varphi^R_{\mathsf{symm}} & \triangleq \forall x, y \cdot R(x,y) \implies R(y,x) & \text{(symmetry)} \end{array} \quad (1)$$

We show that verification is decidable modulo these axioms using a technique that we call *program instrumentation*. Let us fix a relation $R$ and an axiom $\varphi^R_p$, where $p \in \{\mathsf{refl}, \mathsf{irref}, \mathsf{symm}\}$. The idea is to find a function (in fact, a string homomorphism) $h^R_p$ such that for any program $P$, $P$ is correct/coherent modulo $\{\varphi^R_p\}$ iff $h^R_p(\mathsf{Exec}(P))$ is correct/coherent modulo the empty axiom set. Decidability then follows by exploiting the results of [26]. The function $h^R_p$ will capture the properties of the axiom it is trying to eliminate, and so it will be different for different axioms. We first outline these function $h^R_p$, then state their property and prove the decidability result.

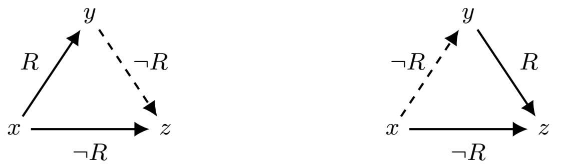

**Fig. 2.** Implied negative relational assumes for a transitive relation $R$. The dashed edges (- - ➞) represent the inferred relationship implied from the relations marked by bold edges (⟶).

For reflexivity, we transform an execution $\rho$ of $P$ to $\rho'$ where $\rho'$ is essentially $\rho$, except that whenever we see the computation of a term, using an assignment of the form "$x := f(\mathbf{z})$", we immediately insert an assume statement that states that $R(x,x)$ holds. More precisely, the homomorphism is defined as,

$$h^R_{\mathsf{refl}}(a) = \begin{cases} a \cdot \text{"}\mathbf{assume}(R(x,x))\text{"} & \text{if } a = \text{"}x := f(\mathbf{z})\text{"} \\ a & \text{otherwise} \end{cases}$$

The homomorphisms used for irreflexivity and symmetry follow similar lines and are outlined in [27].

**Theorem 2.** *For any relation symbol $R$ and $p \in \{\mathit{refl}, \mathit{irref}, \mathit{symm}\}$, the problems of coherent verification modulo $\{\varphi^R_p\}$ and checking coherence modulo $\{\varphi^R_p\}$ are* PSPACE*-complete.*

#### 5.3 Transitivity

We now consider the transitivity axiom for a relation $R$ which says

$$\varphi^R_{\mathsf{trans}} = \forall x, y, z \cdot R(x,y) \wedge R(y,z) \implies R(x,z) \quad \text{(transitivity)} \tag{2}$$

The proof for decidability modulo this axiom is different and more complex that the proofs for reflexivity, irreflexivity, and symmetry. Intuitively, the program instrumentation approach does not seem to work for transitivity. This is because transitivity effects can be global. For example, we may have that the execution asserts the sequence of relational assumes $R(t_1,t_2), R(t_2,t_3), \ldots R(t_{n-1},t_n)$ (here, $t_1, \ldots t_n$ are terms computed by the execution), where some of the intermediate terms may have been dropped by the program (i.e., the variables holding these terms were reassigned). Consequently, relating $t_1$ and (the possibly newly constructed term) $t_n$ requires a principally new machinery. We modify the automaton construction from [26] so that it maintains the transitive closure of the assumptions the program makes. Our main observation is the following:

**Theorem 3.** *Let $\Sigma$ be a first order signature and $V$ a finite set of program variables. Let $\mathcal{A} = \{\varphi^R_{\mathit{trans}} \mid R \in \mathcal{R}_{\mathit{trans}}\}$ for some set of relation symbol $\mathcal{R}_{\mathit{trans}}$ in $\Sigma$. The following observation hold.*

1. *There is a finite automaton* $\mathcal{F}_{trans}$ *(effectively constructable) of size* $O(2^{poly(|V|)})$ *such that for any coherent execution* $\rho$ *that is coherent modulo* $\mathcal{A}$, $\mathcal{F}_{trans}$ *accepts* $\rho$ *iff* $\rho$ *is feasible.*
2. *There is a finite automaton* $\mathcal{C}_{trans}$ *(effectively constructible) of size* $O(2^{poly(|V|)})$ *such that* $L(\mathcal{C}_{trans}) = \mathsf{CoherentExecs}(\Sigma, V, \mathcal{A})$.

*Proof Sketch.* These are in some sense a generalization of the automata constructions used to establish decidability in [26].The automata $\mathcal{F}_{\mathsf{trans}}$ and $\mathcal{C}_{\mathsf{trans}}$ rely on tracking equivalence between values stored in variables, and functional and relational correspondences between these values. However, now since some relations maybe transitive, additional relational correspondences (or their absence) maybe implied for $R \in \mathcal{R}_{\mathsf{trans}}$. The basic idea is to maintain for transitive relations $R$ (a) the transitive closure of the positive relation assumes **assume**$(R(\cdot,\cdot))$, and (b) the negative relational assumes implied by the relational assumes seen in an execution. More precisely, if the execution sees assumes **assume**$(R(x, y))$ and **assume**$(R(y, z))$, then we also add the constraint $R(x, z)$ in the automaton's state. Further, if the execution observes **assume**$(R(x, y))$ and **assume**$(\neg R(x, z))$, then one can infer the constraint $\neg R(y, z)$, and in this case, we accumulate this additional constraint in the state of the automaton. Similarly, if the execution observes **assume**$(R(y, z))$ and **assume**$(\neg R(x, z))$, then one can infer the constraint $\neg R(x, y)$, which is added in the automaton's state. Both these scenarios are illustrated in Fig. 2. A detailed proof is in [27]. □

As a consequence we have the following result.

**Theorem 4.** *For* $\mathcal{A} = \{\varphi^R_{trans} \mid R \in \mathcal{R}_{trans}\}$, *the problems of coherent verification modulo* $\mathcal{A}$ *and checking coherence modulo* $\mathcal{A}$ *are* PSPACE*-complete.*

#### 5.4 Strict Partial Orders

We now turn our attention to axioms that dictate that certain relations be partial or total orders. The anti-symmetry axiom that holds for non-strict orders introduces subtle complications. Recall that $R$ is anti-symmetric if $\forall x, y. R(x, y) \wedge R(y, x) \Rightarrow x = y$; this axiom can imply equality between terms if $R$ holds between a pair of terms. Concretely, if $R$ is anti-symmetric, and the program makes assumptions in an execution that $R(t_1, t_2)$ and $R(t_2, t_1)$ hold, then any model in which such an execution is feasible must also ensure that $t_1 = t_2$. This implicit equality assumption interferes with the notions of coherence and the automata constructions (proofs of the results in [26] and Theorem 4) that compute a congruence closure on terms in a streaming fashion.

Hence, we only consider *strict* partial orders in this section. Recall that a relation $R$ is a strict partial order if it satisfies the irreflexivity axiom and the transitivity axiom, together denoted $\mathcal{A}^R_{\mathsf{SPO}}$. We can prove decidability for problems modulo $\mathcal{A}^R_{\mathsf{SPO}}$ by using our algorithm for irreflexivity and transitivity.

**Theorem 5.** *The following problems are* PSPACE*-complete.*

1. *Given a program* $P$ *that is coherent modulo* $\mathcal{A}^R_{\mathsf{SPO}}$, *determine if* $P$ *is correct.*
2. *Given a program* $P$, *determine if* $P$ *is coherent modulo* $\mathcal{A}^R_{\mathsf{SPO}}$

#### 5.5 Strict Total Orders

A relation $R$ is a strict total order if it is a strict partial order and satisfies:

$$\forall x, y \cdot x \neq y \implies R(x, y) \vee R(y, x) \quad \text{(totality)} \tag{3}$$

Strict total orders are again tricky to handle as the axiom for totality can result in implicit equality between terms. For example, if $\neg R(x, y)$ and $\neg R(y, x)$ then it must be the case that $x = y$. However, if we restrict ourselves to executions that only have assumes of the form **assume**$(R(x, y))$ and do not have any assumes on $\neg R$, i.e., of the form **assume**$(\neg R(x, y))$ then there are no implicit equalities that are entailed.

Unfortunately, in general, program executions can contain negative assumes on $R$ (i.e., assumes of the form **assume**$(\neg R(x, y))$). In order to ensure that executions contain only *positive* assumptions on $R$, we must be careful when identifying executions of programs with conditionals — branches where the assumption $\neg R(x, y)$ holds must be translated to a branch that assumes $R(y, x)$ and a branch that assumes $x = y$. We present a detailed translation in [27].

After such a translation, executions can now have additional equality assumes even if they did not appear in the program. When we refer to coherent programs, we mean that they are coherent according to the above modified notion of executions. This means for such programs to be coherent, all executions must ensure that the additional equality assumes are *early*. And when we talk about coherent verification of programs with total orders, we mean verification for programs that are coherent after this transformation.

We observe that in the absence of any assumes of the form $\neg R(x, y)$ the verification problem modulo strict total orders reduces that modulo strict partial orders, giving us the following ($\mathcal{A}^R_{\mathsf{STO}}$ denote the axioms of irreflexivity, transitivty and totality for the relation $R$).

**Theorem 6.** *The problems of coherent verification, and checking coherence modulo $\mathcal{A}^R_{\mathsf{STO}}$ are* PSPACE*-complete.*

### 6 Axioms Over Functions

We now discuss computational problems modulo axioms that involve function symbols. The treatment of axioms involving functions in the verification of coherent programs is inherently hard. This is because, like in the case of (nonstrict) partial orders and strict total orders, the axioms along with the **assume**-steps in the execution, can imply equalities between terms beyond those entailed by just the **assume** steps in the execution. For example, consider the axiom $\forall x, y \cdot f(x, y) = f(y, x)$ constraining $f$ to be a commutative function. Then terms like $f(f(x, y), z)$ are equal to terms like $f(z, f(x, y))$, and hence when building models we must make sure that functions/relations on such terms are defined in the same way. Terms made equivalent by the functional axioms can be syntactically very different, and keeping track of the equivalence on unbounded executions is hard using finite memory. We consider many natural classes of axioms, and proving both positive and negative results that help delineate the decidability/undecidability boundary.

#### 6.1 Associativity

We now consider the associativity axiom for a function $f$.

$$\varphi^f_{\mathsf{assoc}} \triangleq \forall x, y, z \cdot f(x, f(y, z)) = f(f(x, y), z) \qquad \text{(associativity)} \qquad (4)$$

We show, surprisingly to us, that coherent verification is undecidable modulo $\{\varphi^f_{\mathsf{assoc}}\}$, i.e., even when we have only one axiom that requires only one function to be associative. In fact, the situation is a lot worse — checking the feasibility of even a *single* (even coherent) execution is undecidable, in the presence of a single associative function. The proof of the following result uses a reduction from the word problem for finitely generated semigroups [36].

**Theorem 7.** *Given a a trace $\rho$ that is coherent modulo $\{\varphi^f_{\mathsf{assoc}}\}$, it is undecidable to determine if $\rho$ is feasible. Therefore, the problem checking if a program $P$ that is coherent modulo $\{\varphi^f_{\mathsf{assoc}}\}$ is undecidable.*

#### 6.2 Commutativity

We now consider the commutativity axiom, which is the following

$$\varphi^f_{\mathsf{comm}} \triangleq \forall x, y \cdot f(x, y) = f(y, x) \qquad \text{(commutativity)} \qquad (5)$$

We augment executions with an auxiliary variable $v^* \notin V$ and transform executions using the following homomorphism that uses the auxiliary variable $v^*$

$$h^f_{\mathsf{comm}}(a) = \begin{cases} a \cdot \text{“}v^* := f(y, x)\text{”} \cdot \text{“}\mathbf{assume}(z = v^*)\text{”} & \text{if } a = \text{“}z := f(x, y)\text{”} \\ a & \text{otherwise} \end{cases}$$

We show that the above transformation preserves feasibility and coherence, giving us the following result.

**Theorem 8.** *Verification of coherent programs and checking coherence modulo commutativity axioms is decidable and is* PSPACE*−complete.*

#### 6.3 Idempotence

Next we consider the idempotence axiom for a unary function $f$:

$$\varphi^f_{\mathsf{idem}} \triangleq \forall x \cdot f(x) = f(f(x)) \qquad \text{(idempotence)} \qquad (6)$$

Again, we show that there is a simple homomorphism $h^f_{\mathsf{idem}}$ that preserves coherence and feasibility (see [27]) and reduces verification to one without axioms.

**Theorem 9.** *Verification of coherent programs and checking coherence modulo idempotence axioms is* PSPACE*-complete.*

### 7 Combining Axioms

We have thus far proved decidability results when a relation or functions satisfies certain properties like reflexivity/irreflexivity/symmetry/transitivity or commutativity/idempotence. We now show that all of these results can be combined. That is, we can consider a signature where relations and functions are assumed to satisfy some subset of these properties, and with some being uninterpreted, and the verification problem will remain decidable for coherent programs.

**Theorem 10.** *Let $\mathcal{A}$ be a set of axioms where each relation symbol $R$ is either a total order or satisfies some (possibly empty) subset of properties out of reflexivity, irreflexivity, symmetry, transitivity, and each function symbol $f$ satisfies some (possibly empty) subset out of commutativity and idempotence. The verification problem for coherent programs modulo $\mathcal{A}$ is* PSPACE*-complete.*

The proof of the above result proceeds by *eliminating* axioms one at a time. We first eliminate the relational axioms (reflexivity, irreflexivity, symmetry) in $\mathcal{A}$ using program instrumentation. We then eliminate the functional axioms in $\mathcal{A}$, again using program instrumentation. Our proof relies on this order of elimination of axioms. At this point, the only axioms remaining are those corresponding to transitivity of a subset of relational symbols, which is handled using the automata construction discussed in the proof of Theorem 3.

### 8 Related Work

The theory of equality with uninterpreted functions (EUF) is a widely used theory in many verification applications as it has decidable quantifier free fragment. EUF has been central to advances in verification of microprocessor control [6,4] and hardware verification [1,19] and property directed model checking [18]. EUF has been used as a popular abstraction in software verification [2,3]. Uninterpreted functions have also been studied for equivalence checking and translation validation [35]. Bueno et al [5] demonstrated the effectiveness of uninterpreted programs for verifying SVCOMP benchmarks against control flow properties.

Mathur et al [26] introduced the class of coherent uninterpreted programs and showed that verification of coherent programs, with or without recursive function calls, is a decidable problem. This is one of the few subclasses of program verification over infinite domains that is known to be decidable. Previous works [13,14,31] have established decidability of verification of classes of uninterpreted programs with heavy syntactic restrictions such as disallowing conditionals inside loops or nested loops, etc. As noted in [26], the notion of coherence is close to the notion of a bounded pathwidth decomposition [38]. A term that is created in a coherent execution stays within some program variable (modulo congruence) until the first time all variables containing that term are over-written, and after this point, the execution never computes it again, and thus, the set of windows that contain a term form a contiguous segment of the program execution. Path decomposition and the related notion of tree decomposition have been exploited many times in the literature to give decidability in verification [25,7,8].

The work in [28] extends the work of [26] to *updatable maps* and identifies extensions of coherence that make verification decidable. It utilizes this to provide implementation of verification algorithms for memory safety for a class of heap manipulating programs, including traversal algorithms on data structures such as singly linked list, sorted lists, binary search trees etc. Combining the results of this paper with these results is an interesting future direction.

The class of EPR formulas that consist of universally quantified formulas over relational signatures is a well-known decidable class of first-order logic [37]. EPR-based reasoning has been proved powerful for verification of large-scale systems [33,29,39] and the Ivy [34,30] system is one of the most notable framework that exploits EPR based reasoning for verifying program snippets without recursion. EPR encoding of order axioms such as reflexivity, symmetry, transitivity and total orders has been used in proving programs working over heaps [20].

The work in Kleene Algebra with Tests (KAT) [22] considers problems involving unbounded recursion and choice with abstractions of data, similar to our work. However, while we treat congruence axioms for equality faithfully in our work, it is unclear to us how to express these in KAT or its extensions [21,23,9]. Furthermore, the restrictions of coherence studied in [26] and the work here that are based on bounded path-width notions seem very different from studies of decidable problems in KAT. A study of whether our results can be adapted to yield decidable fragments for KAT is an interesting future direction.

A notable verification technique with an automata-theoretic foundation and that has been very effective in practice is that of trace abstraction due to Heizmann et al [15,16,17,10,11,12]. In this technique, one constructs *iteratively* regular sets that (incompletely) capture the set of all infeasible executions, eventually striving to cover all failing executions of a program, but handling complex theories such as arithmetic. In contrast, our work builds complete automata in one stroke that accept all infeasible traces over a vocabulary, but handles only simple theories with restricted sets of axioms, but yielding decidability. Combining these lines of work for efficient software verification is an interesting future direction.

### 9 Conclusions

By incorporating axioms on functions and relations, decidability results in this paper, enable a more faithfully automatic verification of programs. It is worth noting that the upper bound for all our decidability results is PSPACE, which is the same as that for Boolean programs. Thus, though we consider programs over infinite domains with additional structure, our verification results have the same complexity as that for programs over Boolean domains.

One future direction is to adapt this technique for practical program verification. In this context, adapting our technique within the automata-theoretic technique of [15,17,16,12,10] seems most promising. Second, there are several program verification techniques that use EPR, and in several of these, EPR is used mainly to establish a linear order on the universe [20]. Automatically verifying such programs using our technique is worth exploring.
